# The Clinical Significance of Abnormal Electroencephalography (EEG) Patterns in Patients with Neuropsychiatric Disorders Due to Anti-NMDA Receptor Encephalitis: A Comparative Study

**DOI:** 10.3390/diagnostics15091131

**Published:** 2025-04-29

**Authors:** Alvaro Moreno-Avellán, Arely Juarez-Jaramillo, Maria del Carmen Fernandez Gonzalez-Aragon, Gerardo Quiñones-Pesqueira, Luz Maria Pineda-Centeno, Mariana Espinola-Nadurille, Victoria Martinez-Angeles, Francisco Martinez-Carrillo, Veronica Rivas-Alonso, Daniel San-Juan, Jose Flores-Rivera, Jesus Ramirez-Bermudez

**Affiliations:** 1 The Department of Clinical Neurophysiology, National Institute of Neurology and Neurosurgery, Insurgentes sur 3877, Ciudad de Mexico 14269, Mexico; 2 Neuropsychiatry Unit, National Institute of Neurology and Neurosurgery, Insurgentes sur 3877, Ciudad de Mexico 14269, Mexico; cynthia.arely.6@gmail.com (A.J.-J.); dra.mtz.ang.victoria@gmail.com (V.M.-A.);; 3 Neuroimmunology Clinic, National Institute of Neurology and Neurosurgery, Insurgentes sur 3877, Ciudad de Mexico 14269, Mexico; 4 Epilepsy Clinic, National Institute of Neurology and Neurosurgery, Insurgentes sur 3877, Ciudad de Mexico 14269, Mexico; dsanjuan@innn.edu.mx; 5 The School of Medicine, The National Autonomous University of Mexico, Av. Universidad 3000, Col. Universidad Nacional Autónoma de México, Ciudad de México 04510, Mexico

**Keywords:** anti-NMDA receptor encephalitis, autoimmune encephalitis, autoimmune psychosis, catatonia, EEG, extreme delta brush

## Abstract

**Background:** Anti-NMDA receptor encephalitis is an autoimmune disease characterized by severe neuropsychiatric disturbances, often misdiagnosed as a primary psychiatric disorder. Early diagnosis is crucial, as delayed immunotherapy is associated with worse outcomes. Electroencephalography (EEG) is a widely available tool for detecting abnormalities that may aid in early detection of cases that should undergo a thorough approach. Although EEG has high sensitivity, its specificity remains a challenge. **Methods:** This case-control study was carried out in the National Institute of Neurology and Neurosurgery of Mexico and included 241 patients with acute or subacute neuropsychiatric disturbances, raising the suspicion of autoimmune encephalitis and leading to the determination of NMDA receptor antibodies in the cerebrospinal fluid (CSF). EEG patterns were analyzed to determine the frequency of abnormal findings and their diagnostic value. **Results:** 140 patients were diagnosed as having definite anti-NMDA receptor encephalitis, whereas 101 had a negative determination of NMDA receptor antibodies. Psychosis was very frequent in both groups. However, severe cognitive dysfunction and catatonia were significantly more frequent in anti-NMDA receptor encephalitis patients. EEG abnormalities were significantly more frequent in patients with anti-NMDA receptor encephalitis patients (87.2% vs. 61.2%, *p* < 0.001). Diffuse slowing (75.7% vs. 46.6%, *p* < 0.001) and the extreme delta brush pattern (8.8% vs. 0%, OR = 20.6, *p* = 0.002) were significantly associated with anti-NMDA receptor encephalitis. Logistic regression analysis confirmed that an abnormal EEG remained strongly associated with anti-NMDA receptor encephalitis after adjusting for confounders. **Conclusions:** EEG abnormalities, particularly diffuse slowing and the extreme delta brush pattern, provide important diagnostic clues in patients with a clinical suspicion of anti-NMDA receptor encephalitis. While EEG has high sensitivity, its specificity is enhanced by recognizing distinct patterns. These findings support the integration of EEG into diagnostic algorithms to guide early detection and management of autoimmune encephalitis.

## 1. Introduction

Anti-NMDA receptor encephalitis is a treatable neuroimmunological disease, defined by the presence in cerebrospinal fluid (CSF) of IgG antibodies directed against the NR1 subunit of the NMDA receptor, and causing significant neuropsychiatric disturbances, including psychosis, catatonia, delirium, and dementia [1,2,3]. Besides the cognitive and behavioral manifestations, this immune response causes neurological disturbances such as seizures, abnormal movements, and autonomic abnormalities [1,4,5,6]. Generally speaking, anti-NMDA receptor encephalitis occurs without a prior psychiatric history. However, the onset in most patients involves acute or subacute psychopathological disturbances, before progressing to a stage with unequivocal neurological-like seizures, abnormal movements, and coma states. Thus, psychiatric referrals, misdiagnoses, and treatment delays are frequent [4,5,7]. This is important because delayed immunotherapy is associated with poorer prognosis and higher mortality [8].

Regarding patients with acute or subacute psychiatric disturbances, there is significant debate on how to identify cases with a high likelihood of autoimmune encephalitis. Current evidence suggests that when screening for antineuronal antibodies is performed systematically in all patients with first-episode psychosis—without clear red flags or neurological features of autoimmune encephalitis, such as seizures or dyskinesias—the prevalence of positive CSF findings is low or minimal [9,10]. Therefore, a red flag system for autoimmune encephalitis and specific criteria for possible autoimmune psychosis have been proposed to improve the accuracy of the diagnostic suspicion [11,12,13]. This is important since many cases of autoimmune encephalitis are cared for in mental health services, and lumbar puncture is not available in many of them. If serum testing is carried out without CSF testing, false-positive and false-negative results may lead to misdiagnosis [14,15]. Therefore, it is necessary to have a clear indication for CSF testing in patients with a clinical suspicion of autoimmune encephalitis [5,15,16]. In this context, EEG is considered a valuable tool, because it is generally reported as abnormal in the majority of patients with anti-NMDA receptor encephalitis, with a high sensitivity (96%), although a normal EEG does not exclude anti-NMDARE, especially in the early stages of the disease. In the context of mental health services, EEG is a useful resource due to its high sensitivity and because it is already available in many settings and has reasonable costs. Thus, it has been regarded as an initial paraclinical study allowing for a more thorough diagnostic approach including the CSF analysis via lumbar puncture.

Current research supports the hypothesis that EEG abnormalities are highly sensitive, with limited specificity. However, some considerations are necessary to correct this general concept. Firstly, it has been reported that initial EEG studies may be abnormal only in the minority of cases, although with repeated studies, the large majority of the cases will eventually have a clinically significant abnormality in the EEG [17]. The most common abnormalities are focal (73%) or diffuse (67%) slowing [17]. Some EEG findings have high diagnostic value, like the extreme delta brush pattern. However, although this electrophysiological sign is highly specific, which means that it leads to minimal false positive results, it has a low sensitivity, which means that many patients with definite anti-NMDA receptor encephalitis will not present the sign. In fact, the sign is only present in 11–14% of the cases, mostly in severely affected patients [17,18,19,20]. A systematic review of EEG findings in anti-NMDA receptor encephalitis, including 446 EEG cases, showed that abnormal EEGs were present in 83.6% of the cases; the main findings were diffuse encephalopathy in 60.3%, delta range slowing in 18.1%, and epileptiform abnormalities (including sharp waves, PLEDs, GPEDs) in 15.02%. Electrographic seizures were documented in 8.74%. The extreme delta brush pattern was observed in 6.73%, generalized rhythmic delta activity in 4.49%, diffuse beta activity in 2.91%, and status epilepticus was observed in 2.91% [21].

## 2. Materials and Methods

### 2.1. Design, Selection Criteria, and Sampling

We conducted a comparative, case-control study (registry 86/19, approved by the Institutional Research and Ethics Committees) at the National Institute of Neurology and Neurosurgery of Mexico (NINN). Patients were hospitalized in the NINN during 2016–2024. The following criteria were used for selection in the study: Patients without a previous history of psychiatric disorders were included if they met the following conditions: (1) They exhibited a clinically significant onset of neuropsychiatric signs and symptoms (i.e., patterns of psychosis, delirium, catatonia, or affective syndromes); and (2) They showed at least one red flag suggesting a possible diagnosis of autoimmune encephalitis, such as: (a) seizures, (b) abnormal movements, (c) catatonic signs, (d) severe and disproportionate cognitive disturbance, or (e) clinical worsening following the use of antipsychotics, particularly with features suggestive of neuroleptic malignant syndrome. [1,11] A consecutive case sampling was used according to the elegibility criteria.

### 2.2. Clinical and Paraclinical Measurments

The diagnostic approach included a careful psychopathological and neurological examination. All patients were assessed through paraclinical diagnostic studies, including EEG, MRI, ^18F-^FDG PET/CT, and cerebrospinal fluid (CSF) analysis via lumbar puncture. A determination of NMDA receptor antibodies in the CSF was carried out in all patients. Graus et al. criteria were used to classify patients as having or not having a definitive anti-NMDAR diagnosis, including the reasonable exclusion of other causes [22]. A systematic evaluation was carried out to register the psychopathological, neurological, and paraclinical features of each case, as described elsewhere [1]. The modified Rankin scale (mRS) [23] was used to register the functional state of each patient at admission and discharge, considering that this instrument has been widely used in research programs of autoimmune encephalitis [8]. The Montreal Cognitive Assessment test was used to assess cognitive performance [24,25] by using the standard categorical approach: patients were classified as having a mild (25–18 points), moderate (17–10 points), or severe dysfunction (9–0 points) [24]. A category for “not being able to complete the test” was considered as the most severe rank.

### 2.3. Electroencephalography (EEG) Data Acquisition and Interpretation

Scalp electroencephalography (EEG) was performed in a routine manner within the first 24 to 72 h after hospital admission, and before the use of immunotherapy in patients that received that intervention. EEG recordings were obtained following the international 10–20 system. Each recording lasted between 15 and 30 min, in accordance with the guidelines of the American Clinical Neurophysiology Society. We used the NicoletOne hardware (Pleasanton, CA, USA) and The EB Neuro SpA Software Galileo, version Gal.Net 10.0 (Florence, Italy). Impedances were maintained below 5 mOhms, and recordings were performed with gold cup electrodes, a sensitivity setting of 7 mm/second, and filters ranging from 1 to 70 Hz. EEGs were interpreted using an anteroposterior bipolar longitudinal montage, including double banana, center-parasagittal, bipolar transversal, and monopolar referential montages. The EEGs were interpreted according to the terminology and definitions established by the Committee on Terminology of the International Federation of Societies for EEG and Clinical Neurophysiology [26,27,28,29]. The EEG findings were classified according to the following abnormal patterns: (A) Presence of generalized or focal slowing. The severity of generalized or focal slowing was categorized as mild (6–7 Hz), moderate (4–5 Hz), or severe (≤3 Hz). (B) Generalized rhythmic delta activity. (C) Extreme delta brush, defined as rhythmic delta activity from 1 to 3 Hz with superimposed bursts of rhythmic 20 to 30 Hz beta frequency activity “riding” on each delta wave. (D) Electrographic seizures, defined as abnormal paroxysmal events distinct from the background, lasting longer than 10 s (or shorter if associated with a clinical change), and exhibiting temporal–spatial evolution in morphology, frequency, and amplitude, with a plausible electrographic field. (F) Interictal epileptiform discharges [26,27,28,29]. The EEG interpretation was carried out before knowing the results of the antibody determination in the CSF.

### 2.4. Data Analysis

Statistical analysis was performed with SPSS package version 25. Initially, we obtained descriptive statistics. The Kolmogorov-Smirnov normality test was used to analyze the distribution of continuous variables. To compare the groups with and without a definite diagnosis of anti-NMDA receptor encephalitis, we used Pearson’s chi-square for categorical variables, as well as T-student or Mann-Whitney tests for continuous variables, based on their distribution. Following the recommendations of the Equator network, we used the STARD guidelines 2015 checklist for diagnostic studies to calculate several measures of diagnostic validity for the main EEG findings like the sensitivity, the specificity, the positive and negative likelihood ratios, as well as the overall accuracy calculated by using the standard equations [30]. Finally, we used a logistic regression model to control the role of confounding variables that could potentially modify the EEG results, for instance, the use of lorazepam and antipsychotics.

## 3. Results

### 3.1. General Characteristics of the Sample

In total, 265 patients were assessed during the 2016–2024 period, and 241 were included in the study as they fulfilled the selection criteria. The mean age was 31.2 years (SD 13.1) including 49.4% female (*n* = 119). One-hundred and forty patients had a definite anti-NMDA receptor encephalitis, and 101 had a negative determination of NMDA receptor antibodies in the CSF. Within this group, the final diagnoses were established: (a) fifty-seven patients had a final diagnosis of probable autoimmune encephalitis, with negative NMDAR antibodies, (b) twenty-one patients were classified as having a primary psychotic disorder, (c) five patients had a final diagnosis of viral encephalitis, (d) four patients had a final diagnosis of epilepsy, (e) four patients had a final diagnosis of prion disease, (f) three patients had neuropsychiatric systemic lupus erythematosus, (g) three patients had a final diagnosis of bacterial meningoencephalitis, (h) two patients had anti-LGI1 encephalitis, (i) one patient had COVID-19 encephalopathy, and (j) one patient had metabolic encephalopathy. This was regarded as the comparison group for the analysis.

### 3.2. Comparative Analysis of Patients With and Without a Final Diagnosis of Anti-NMDA Receptor Encephalitis

The analysis of demographic variables showed that patients with anti-NMDA receptor encephalitis were significantly younger (28.4 years +/− 10.2 vs. 35.3 years 3 +/− 14.4, *p* < 0.001, *t*-student test). We did not find a higher frequency of female patients (45.7% vs. 54.5%, *p* = 0.181, chi-square test). Table 1 shows the clinical features of the sample. As may be seen, psychosis was present in most patients in both groups. Some psychopathological features were significantly more frequent in patients with anti-NMDA receptor encephalitis, namely, (a) a severe and disproportionate cognitive dysfunction (*p* = 0.009, chi-square test), and (b) a diagnosis of delirium (*p* = 0.020, chi-square test), a diagnosis of catatonia (*p* = 0.002, chi-square test). Also, some neurological signs were more frequent in patients with anti-NMDA receptor encephalitis, namely, (a) seizures (*p* < 0.001, chi-square test), (b) status epilepticus (*p* = 0.003, chi-square test), (c) abnormal movements (*p* = 0.004, chi-square test), and (d) autonomic abnormalities (*p* < 0.001, chi-square test).

### 3.3. The Diagnostic Value of Electroencephalography (EEG) Patterns in Patients With and Without a Final Diagnosis of Anti-NMDA Receptor Encephalitis

As may be seen in Table 2, an abnormal EEG was more frequent in patients with anti-NMDA receptor encephalitis vs. the comparison group (87.2% vs. 61.2%, *p* < 0.001, chi-square test). Table 2 presents the measures of diagnostic validity for each of the main EEG patterns, including sensitivity, specificity, positive and negative likelihood ratios, and overall accuracy. In the case of the main result of the study, an abnormal EEG had a sensitivity of 85.71% (95% CI, 78.97–90.56%), and a specificity of 57.43 (95% CI, 47.69–66.62%). Also, two patterns were significantly higher in patients with anti-NMDA receptor encephalitis: (a) diffuse slowing (75.7% vs. 46.6%, *p* < 0.001, chi-square test), and (b) the extreme delta brush pattern (8.8% vs. 0%, *p* = 0.002), which was significantly related to the length of the hospitalization (96.6 +/− 65.16 days in the patients with anti-NMDAR encephalitis vs. 39.51 +/− 28.20 days in the comparison group, *p* < 0.001, *t*-test).

In our sample, EEG demonstrated a sensitivity of 85.7% and a specificity of 42.6%, with an overall accuracy of 67.6%. Regarding CSF analysis (based on standard parameters including cell count, protein, and glucose levels), the sensitivity of detecting abnormal results was 58.0%, the specificity was 60.2%, and the overall accuracy was 58.8%. For MRI (considering conventional structural sequences), the sensitivity of detecting abnormalities was 56.9%, the specificity was 64.4%, and the accuracy was 59.7%.

Although this study was not designed to systematically record the clinical course longitudinally, we did collect some outcome data during hospitalization that were analyzed in relation to EEG patterns: (A) Patients who developed status epilepticus had a higher proportion of abnormal EEGs at the beginning of hospitalization compared to those who did not develop status epilepticus (100% vs. 69.2%, *p* < 0.001, Pearson’s chi-square test). (B) Patients who developed stupor or coma during hospitalization had a higher proportion of abnormal EEGs than those who did not (94.4% vs. 70.2%, *p* = 0.002, Pearson’s chi-square test). (C) Patients who required admission to the Intensive Care Unit had a higher proportion of abnormal EEGs at the beginning compared to those who did not require ICU admission (88.2% vs. 70%, *p* = 0.009). (D) Patients who died during hospitalization (*n* = 12) had a higher proportion of interictal epileptiform discharges at the beginning compared to those who survived (50% vs. 14%, *p* = 0.001).

Table 3 shows the results of a logistic regression model estimating the value of an abnormal EEG in patients with and without a definite diagnosis of anti-NMDA receptor encephalitis, adjusted for confounding variables. We included age, because this variable was clearly different among the groups, as well as an abnormal result of the CSF cytochemical analysis (pleocytosis), because this variable was related to an abnormal EEG in our sample. Finally, we included the use of lorazepam, and the use of antipsychotics, as these pharmacological agents are frequently used in patients with neuropsychiatric disturbances, and they can modify the results of the EEG in some patients. However, in our logistic regression model, an abnormal EEG was significantly related to the definite diagnosis of anti-NMDA receptor encephalitis after adjusting for these potentially confounding variables, with an OR of 4.20 (CI 95% 2.06–8.54), which is highly significant.

### 3.4. The Relationship Between Electroencephalography (EEG) Patterns and Neuropsychiatric Clinical Patterns

We found several associations between EEG patterns and neuropsychiatric symptoms in the total sample of 241 cases, as follows: (A) Patients with severe cognitive dysfunction at admission showed a higher proportion of abnormal EEGs compared to those without cognitive dysfunction (78.5% vs. 64.1%, *p* = 0.0017, Pearson’s chi-square test). (B) Patients who developed delirium during the hospitalization also exhibited a higher proportion of abnormal EEGs compared to those without delirium (79.2% vs. 64.4%, *p* = 0.012, Pearson’s chi-square test).

## 4. Discussion

**Figure 1:**
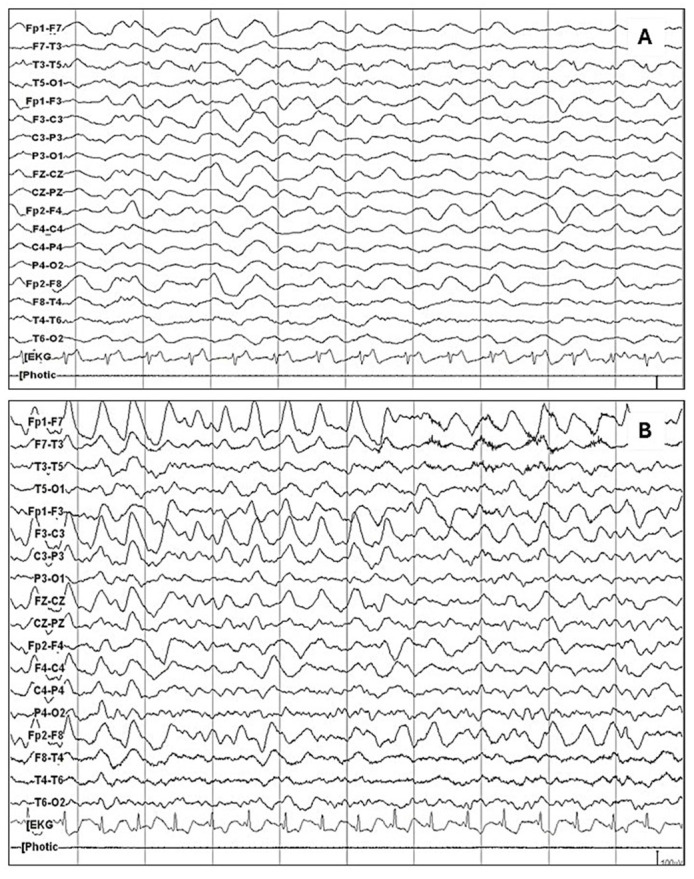
The most common abnormal pattern in patients with anti-NMDAR encephalitis was the diffuse slowing of the baseline rhythm, as observed in the following examples: (**A**) Generalized delta activity from 1.5 to 2 Hertz is observed. (**B**) A delta–theta rhythm with left frontal predominance is observed.

**Figure 2:**
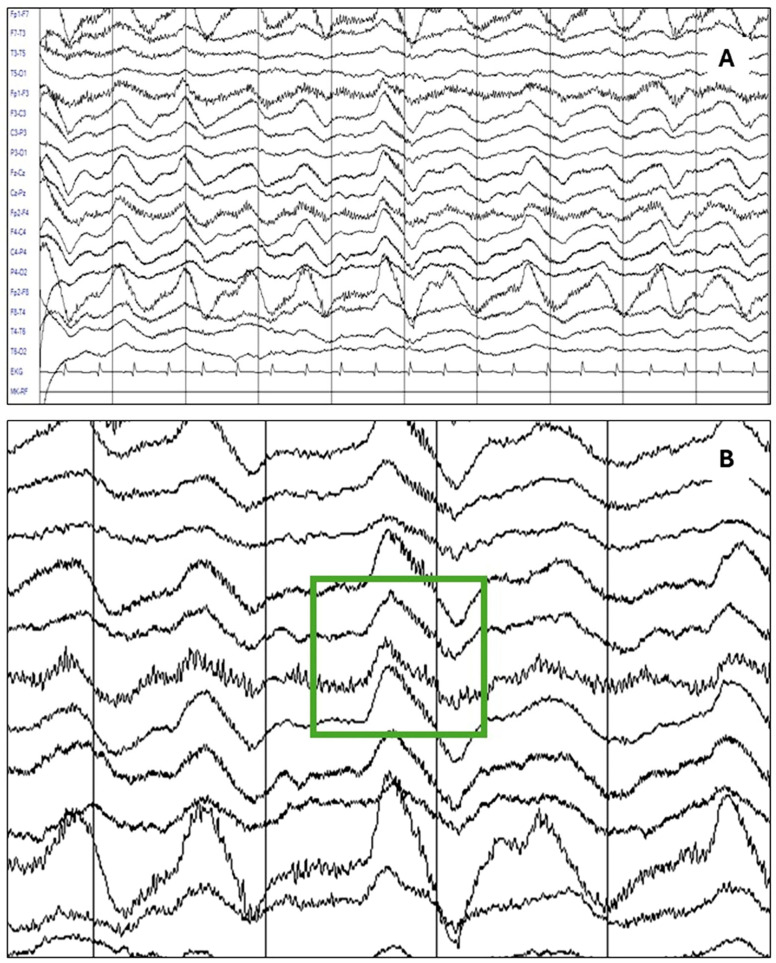
The most specific abnormality in patients with anti-NMDAR encephalitis was the extreme delta brush pattern, as seen in the following example: (**A**) An EEG study of a patient with a definite diagnosis of anti-NMDAR encephalitis showing the delta brush pattern. (**B**) A detailed view of the same study highlighting the rhythmic delta activity (RDA) at 1.5 Hertz with superimposed bursts of rhythmic beta-frequency activity (25 to 30 Hz).

The most relevant findings of this study may be summarized as follows: in our sample of 241 patients with acute and subacute neuropsychiatric disturbances, under suspicion of autoimmune encephalitis due to the presence of red flags, 140 received a definite diagnosis of anti-NMDA receptor encephalitis, whereas 101 had a negative determination of NMDA receptor antibodies and were used as a comparison group. EEG abnormalities were present in 85% of the patients with anti-NMDA receptor encephalitis patients, with a 95% confidence interval of 78.96–90.56%. This frequency was significantly higher compared to the comparison group. Diffuse slowing (75.7%) and the extreme delta brush pattern (8.8%) were significantly associated with anti-NMDARE. Logistic regression analysis confirmed that an abnormal EEG remained strongly predictive of anti-NMDARE after adjusting for confounders. As a resource to improve the recognition of these patterns, Figure 1 presents the main EEG patterns through graphical examples. Figure 1A shows the study of a patient in which a pattern of generalized delta activity from 1.5 to 2 Hertz was observed, whereas Figure 1B shows the study of a patient in which a delta–theta rhythm with left frontal predominance is observed. Figure 2 depicts the extreme delta brush pattern.

The sensitivity of abnormal EEG findings was 85.71% and the specificity was 57.43. Our results are consistent with previous research. According to the systematic review of 446 patients by Gillinder et al., an abnormal EEG was present in 83.6% of the cases [21]. This is clearly within our confidence interval. In our study, the most common pattern was diffuse slowing. This is consistent with the systematic review by Gillinder et al., in which diffuse encephalopathy was the most common abnormality, although the frequency was 75.7% in our sample, whereas the frequency in the systematic review was 60.3%. The frequency of interictal epileptiform discharges was 16.4% in our study, consistent with the results of the systematic review, which was 15.02%. In our study, the specificity of the extreme delta brush pattern was 100%. However, the sensitivity is low, as it was present only in 7.1% of the cases of anti-NMDA receptor encephalitis. The frequency is quite similar to the rate of 6.73% observed in the systematic review [21].

Clinicians should not rely on the absence of the delta brush pattern to rule out anti-NMDA receptor encephalitis. However, given its low sensitivity, it occurs in only 5.9–33.3% of cases [31]. However, its high specificity makes it a valuable diagnostic indicator when present. Since CSF antibody testing is sometimes unavailable or delayed, the presence of the delta brush pattern—in the appropriate clinical context—can significantly increase diagnostic confidence and support the early initiation of targeted treatment. We also observed a significant relationship between the extreme delta brush pattern and the length of the hospitalization, and with the need for ICU care. It has been observed that this pattern is a measure of the severity of the illness. For instance, a multicentric study found that an extreme delta brush pattern was significantly linked to recurrent seizures, status epilepticus, and the need for aggressive treatment (induced coma and ICU care) [32]. Also, in a previous study of 98 Chinese anti-NMDAR encephalitis patients (79 acute, 39 recovery), more severe EEG abnormalities correlated with worse clinical symptoms (impaired consciousness, movement disorders, coma) and longer hospital/ICU stays (*p* < 0.05) [33].

Since the initial characterization of the extreme delta brush (EDB) pattern in patients with anti-NMDAR encephalitis and its association with longer hospital stays, several case series have sought to identify reliable EEG biomarkers for prognosis, including findings such as the absence of parietal or temporal epileptiform activity. Additionally, the lower modified Rankin scale at admission may be associated with shorter hospitalizations [18,34]. Compared to patients with other types of encephalitis, patients with anti-NMDAR encephalitis exhibited significantly greater delta peak power, elevated beta/alpha and Gamma/beta frequency ratios, and reduced alpha and beta peak power as well as a lower beta/delta frequency ratio at the first EEG. Notably, higher delta and alpha peak power were associated with poorer clinical outcomes at discharge and follow-up, whereas increased Gamma peak power correlated with more favorable outcomes [35].

In our sample, EEG emerged as the diagnostic tool with the highest sensitivity and accuracy. However, given that CSF analysis and structural MRI exhibit higher specificity, we believe that a multimodal diagnostic approach may enhance overall diagnostic accuracy.

### Limitations

Among the limitations of our study, we should note that the EEG patterns are dynamic and closely linked to the stage of the disease. As we aimed to describe the initial EEG recordings at the beginning of the hospitalization, and before the use of immunotherapy, it is possible that some patients could present further abnormalities during the advanced periods of the hospitalization, for instance, in the ICU. It is likely that this approach leads to a systematic underestimation of the sensitivity of EEG patterns in the diagnosis of anti-NMDA receptor encephalitis. For instance, the frequency of the delta brush pattern and status epilepticus patterns may have been higher in patients admitted to the ICU. However, this design also offers the advantage of providing a more homogeneous sample, highlighting the features of patients at admission, during the early stages of their clinical course, when relevant diagnostic and therapeutic decisions must be made. We are looking forward to make a more longitudinal description of the EEG patterns through repeated measurements. However, Moise A et al. 2021 in a retrospective cohort study of 64 patients (27% *n* = 17 patients with anti-NMDA receptor encephalitis) with autoimmune encephalitis who underwent at least 6 h of continuous EEG monitoring, found that only the extreme delta brush pattern is specifically related to anti-NMDA receptor encephalitis and all patients showed abnormal EEG findings; unfortunately, the number of patients with anti-NMDA receptor encephalitis is small and the time of continuous EEG monitoring is limited [36]. Also, our study was not designed to measure the treatment response; therefore, we were not able to analyze the relationship between the EEG patterns and the treatment response. From a methodological perspective, the lack of inter-rater reliability measurement is a limitation of this study. Another limitation refers to pharmacological confounding. As most patients receive antiseizure medications, antipsychotics, and lorazepam for the control of catatonic signs, we have few drug-naïve patients. Although this variable introduces a source of heterogeneity into the sample, we believe it is valuable to provide data that more closely reflects real-world clinical settings, as it allows for clinical judgment in patients who are receiving medication—which is likely the case in most clinical contexts. Therefore, our tactic was to control this source of confounding through a multivariate analysis. Finally, the statistical stability of the diagnostic measures (sensitivity, specificity, likelihood ratios) is typically acquired with larger samples. We hope that our study will contribute to the precision of further meta-analysis.

## 5. Conclusions

These findings support the integration of EEG into diagnostic algorithms to guide early detection and management of autoimmune encephalitis. From our perspective, this is particularly useful in the context of mental health services and general hospitals, where specialized diagnostic methods, including the CSF determination of NMDA receptor antibodies, via lumbar puncture, are not always available. Two conclusions follow from this analysis: (1) Considering the sensitivity of an abnormal EEG in patients with acute and subacute neuropsychiatric disturbances had red flags, this finding can be used to improve the selection of patients in which a CSF analysis is relevant. Diffuse slowing was the most common finding, and it supports the referral to a neurological service or a neurological consultation, especially if delta range slowing is observed. (2) Considering the specificity of the extreme delta brush pattern in patients with anti-NMDA receptor, this EEG finding indicates the need for a specialized consultation to deliver immunotherapy in patients with a clinical pattern consistent with autoimmune encephalitis.

## Figures and Tables

**Table 1:** A comparative analysis of the clinical patterns observed in patients with a definite diagnosis of anti-NMDA receptor encephalitis vs. patients with a negative determination of NMDA receptor antibodies in the cerebrospinal fluid.

Clinical Pattern	Patients with Anti-NMDAR Encephalitis (*n* = 140)	Patients with a Negative Determination of NMDAR Antibodies (*n* = 101)	OR (CI 95%)	*p* Value
**Psychopathological Features**				
Psychosis	111 (79.3%)	77 (76.2%)	1.19 (0.64–2.20)	0.573
Severe Cognitive Dysfunction	104 (74.3%)	59 (58.4%)	2.05 (1.18–3.55)	0.009
Delirium	98 (70%)	56 (55.4%)	1.87 (1.10–3.19)	0.020
Psychomotor Agitation	94 (67.1)	59 (58.4%)	1.45 (0.85–2.47)	0.165
Catatonia	86 (61.4%)	42 (41.6%)	2.23 (1.32–3.77)	0.002 *
Anxiety	76 (54.3%)	50 (49.5%)	1.21 (0.72–2.02)	0.463
Mania	27 (19.3%)	23 (22.8%)	0.810 (0.43–1.51)	0.510
Depression	25 (17.8%)	22 (21.8%)	0.78 (0.41–1.48)	0.448
**Neurological Features**				
Seizures	82 (58.6%)	25 (24.8%)	4.29 (2.44–7.54)	<0.001 *
Status Epilepticus	29 (20.7%)	7 (6.9%)	3.50 (1.47–8.37)	0.003 *
Abnormal Movements	82 (58.6%)	40 39.6%)	2.15 (1.28–3.63)	0.004
Autonomic Abnormalities	76 (54.3%)	29 (28.7%)	2.94 (1.71–5.08)	<0.001 *

* Pearson’s chi-square test. Significant after Bonferroni correction for multiple comparisons (*p* < 0.05/6 = 0.004).

**Table 2:** A comparative analysis of the EEG patterns observed in patients with a definite diagnosis of anti-NMDA receptor encephalitis vs. patients with a negative determination of NMDA receptor antibodies in the cerebrospinal fluid.

EEG Variable	Patients with Anti-NMDAR Encephalitis (*n* = 140)	Patients with a Negative Determination of NMDAR Antibodies (*n* = 101)	Diagnostic Values	*p* Value
Abnormal EEG	120 (85.7%)	58 (57.4%)	Sensitivity: 85.7% Specificity: 42.6% Positive LR: 1.49 Negative LR: 0.34 Accuracy: 0.67	<0.001
Diffuse Slowing	105 (75.0%)	44 (43.6%)	Sensitivity: 75% Specificity: 56.4% Positive LR: 1.72 Negative LR: 0.44 Accuracy: 0.67	<0.001
Focal Slowing	12 (8.6%)	8 (7.9%)	Sensitivity: 8.6% Specificity: 92.1% Positive LR: 1.09 Negative LR: 0.99 Accuracy: 0.43	0.857
Interictal Epileptiform Discharges	23 (16.4%)	15 (14.9%)	Sensitivity: 16.4% Specificity: 85.1% Positive LR: 1.1 Negative LR: 0.98 Accuracy: 0.45	0.740
Interhemispheric Asymmetric	23 (16.4%)	9 (8.9%)	Sensitivity: 16.4% Specificity: 91.1% Positive LR: 1.84 Negative LR: 0.92 Accuracy: 0.47	0.090
Extreme Delta Brush Pattern	10 (7.1%)	0 (0%)	Sensitivity: 7.1% Specificity: 100% Positive lr: infinity Negative LR: 0.93 Accuracy: 0.46	0.006

**Table 3:** Logistic regression model estimating the value of an abnormal EEG in patients with and without a definite diagnosis of anti-NMDA receptor encephalitis, adjusted for confounding variables.

Variable	*p*	OR (CI 95%)
Age	<0.001	0.95 (0.92–0.97)
Abnormal CSF (Pleocytosis)	0.009	2.23 (1.22–4.07)
Use of Lorazepam	0.062	2.03 (0.96–4.27)
Use of Antipsychotics	0.153	0.56 (0.26–1.23)
Abnormal EEG	<0.001	4.55 (2.27–9.13)

## Data Availability

The research data are available upon request to the corresponding author.

## Abbreviations

The following abbreviations are used in this manuscript:

EDB	Extreme Delta Brush
EEG	Electroencephalogram
NMDA	*N*-Methyl-D-Aspartate
